# High Beta-Palmitate Fat Controls the Intestinal Inflammatory Response and Limits Intestinal Damage in Mucin Muc2 Deficient Mice

**DOI:** 10.1371/journal.pone.0065878

**Published:** 2013-06-12

**Authors:** Peng Lu, Fabiana Bar-Yoseph, Liora Levi, Yael Lifshitz, Janneke Witte-Bouma, Adrianus C. J. M. de Bruijn, Anita M. Korteland-van Male, Johannes B. van Goudoever, Ingrid B. Renes

**Affiliations:** 1 Division of Neonatology, Department of Pediatrics, Erasmus MC-Sophia, Rotterdam, the Netherlands; 2 Department of Pediatrics, Emma Children’s Hospital - AMC, Amsterdam, the Netherlands; 3 Enzymotec LTD, Kfar Baruch, Israel; 4 Department of Pediatrics, VU University Medical Center, Amsterdam, the Netherlands; Inserm, France

## Abstract

**Background:**

Palmitic-acid esterified to the sn-1,3 positions of the glycerol backbone (alpha, alpha’-palmitate), the predominant palmitate conformation in regular infant formula fat, is poorly absorbed and might cause abdominal discomfort. In contrast, palmitic-acid esterified to the sn-2 position (beta-palmitate), the main palmitate conformation in human milk fat, is well absorbed. The aim of the present study was to examine the influence of high alpha, alpha’-palmitate fat (HAPF) diet and high beta-palmitate fat (HBPF) diet on colitis development in Muc2 deficient (Muc2^−/−^) mice, a well-described animal model for spontaneous enterocolitis due to the lack of a protective mucus layer.

**Methods:**

Muc2^−/−^ mice received AIN-93G reference diet, HAPF diet or HBPF diet for 5 weeks after weaning. Clinical symptoms, intestinal morphology and inflammation in the distal colon were analyzed.

**Results:**

Both HBPF diet and AIN-93G diet limited the extent of intestinal erosions and morphological damage in Muc2^−/−^ mice compared with HAPF diet. In addition, the immunosuppressive regulatory T (Treg) cell response as demonstrated by the up-regulation of *Foxp3*, *Tgfb1* and *Ebi3* gene expression levels was enhanced by HBPF diet compared with AIN-93G and HAPF diets. HBPF diet also increased the gene expression of *Pparg* and enzymatic antioxidants (*Sod1*, *Sod3* and *Gpx1*), genes all reported to be involved in promoting an immunosuppressive Treg cell response and to protect against colitis.

**Conclusions:**

This study shows for the first time that HBPF diet limits the intestinal mucosal damage and controls the inflammatory response in Muc2^−/−^ mice by inducing an immunosuppressive Treg cell response.

## Introduction

Human milk provides the best nourishment for full-term infants, by which approximately half of the dietary calories are supplied as fat [1]. More than 98% of milk fat is in the form of triglycerides containing fatty acids esterified to glycerol, with palmitic acid (C16∶0) representing about 20–25% of total milk fatty acid. In human milk, palmitic acid is predominantly esterified to the sn-2 position of the triglyceride (beta-palmitate) [2]. However, in vegetable oils, the main constituents of infant formula fat, the palmitic acid is predominantly esterified to the sn-1,3 positions of the triglycerides (alpha, alpha’-palmitate) [3]. Structured triglycerides (synthetic beta-palmitate) are synthesized through enzymatic processing, whereby a large amount of alpha, alpha’-palmitate fat is converted to beta-palmitate fat [4], [5]. It has been reported that high beta-palmitate fat (HBPF) rather than high alpha, alpha’-palmitate fat (HAPF) enables easy digestion and absorption of fatty acid [6], [7], [8], [9] and calcium [10]. Moreover, HBPF was shown to enhance the proliferation of spleen lymphocytes [11]. However, the influence of HBPF on intestinal damage remains unknown.

Mucins are the principal components of the intestinal mucus layer [12], which forms a physical barrier protecting the underlying epithelium against luminal substances and microbes [13], [14], [15]. Deficiency of Muc2 affects the protective capacities of the mucus layer [16], and as a consequence, bacteria are in direct contact with the intestinal epithelial cells [17]. This in its turn leads to the development of spontaneous colitis in Muc2^−/−^ mice, a well-described animal model for enterocolitis [18], [19], [20]. In this model, colonic mucosal damage is not observed before weaning when immunosuppressive regulatory T (Treg) cells dominate the immune response. Interestingly, after weaning the Treg cell response declines and mucosal damage appears [21]. These data suggest that components of human milk might be able to limit intestinal inflammation and beta-palmitate could be such a component. Furthermore, increasing evidence has shown a protective role of Treg cells in inflammatory diseases [22], and patients with inflammatory bowel diseases (IBD) have reduced Treg cell numbers compared with patients with non-IBD inflammatory diseases [23].

The present study was designed to investigate the influence of alpha, alpha’-palmitate fat and beta-palmitate fat on colitis development in Muc2^−/−^ mice. We hypothesized that HBPF diet, which mimics the fat composition and properties of human milk fat, limits the intestinal mucosal damage and controls the inflammatory response in Muc2^−/−^ mice. Therefore, Muc2^−/−^ mice were fed AIN-93G reference diet [24], HAPF diet or HBPF diet for 5 weeks after weaning, and clinical symptoms and intestinal damage and inflammation were analyzed.

## Methods

### Animals

The 129Sv-Muc2^−/−^ mice were generated from Muc2 heterozygous mice as previously described [19]. All mice were housed in the same specific pathogen-free environment with free access to acidified tap water in a 12-hour light/dark cycle. All animal experiments were reviewed by and performed with approval of the Erasmus MC Animal Ethics Committee (approval number: EMC 2087), Rotterdam, the Netherlands. All mice were tested negative for *Helicobacter hepaticus* and norovirus infection.

### Experimental Setup

Muc2^−/−^ mice were divided into three diet groups which only differed in fat compositions: standard AIN-93G diet as a reference group, HAPF diet and HBPF (InFat™, Advanced Lipids AB) diet containing 11.1%, 16.7% and 16.8% total palmitic acid, with 6.3%, 11.0% and 50.4% of the palmitic acid esterified to the sn-2 position, respectively (see Table 1 for more detailed information on fatty acid composition). Three male and 3 female mice were included in each group. All three diets were prepared by Research Diet Services, Wijk bij Duurstede, the Netherlands. Animals were weaned from mother’s milk at the age of 23 or 24 days, housed separately, and received one of the above described diets *Ad libitum* for 5 weeks.

**Table 1:** Fatty acid composition in the diets.

	AIN-93G	HAPF	HBPF
**Fatty acid composition (%)**			
C8	nd	nd	nd
C10	nd	nd	nd
C12	nd	nd	0.2
C14	nd	0.3	0.2
C15	nd	nd	nd
C16	11.1	16.7	16.8
C16∶1	0.1	0.2	0.1
C17	nd	nd	nd
C18	4.5	3.4	3.8
C18∶1	24.7	36	42.2
C18∶2 (n-6)	51.6	40.3	34
C18∶3 (n-3)	6.6	1.8	1.5
C20	0.4	0.3	0.3
C20∶1	0.2	0.3	0.3
C22	0.4	0.4	0.4
C22∶1	0.3	0.1	0.1
C24	0.1	0.2	0.1
C16 sn-2	2.1	5.5	25.4
C16 sn-2/total C16 (%)	6.3	11.0	50.4
Total fat content/diet (%)	7.2	7.2	7.1

nd: not detected.

The food intake and body weight were recorded each week. Animals were sacrificed at the end of experiment, and colonic tissue samples were excised immediately and either fixed in 4% (w/v) paraformaldehyde in phosphate-buffered saline or stored in RNAlater (Qiagen, Venlo, the Netherlands) at −20°C.

### Histology and Immunohistochemistry

Paraformaldehyde fixed colonic tissues were embedded in paraffin, and 4-µm-thick sections were stained with hematoxylin and eosin to study histologic changes as described previously [25]. The erosion score was assessed as follows: 0, no erosions; 1, 0–25% of the epithelium is erosive; 2, 25–50% of the epithelium is erosive; 3, 50–75% of the epithelium is erosive; 4, 75–100% of the epithelium is erosive; 5, 100% erosions (i.e. no epithelium present). To detect differences in mucosal and epithelial thickness in the colon, 5 to 10 well-oriented crypts were chosen per intestinal segment and measured using calibrated Leica Image Manager 500 software (Leica Microsystems, Rijswijk, the Netherlands). All data were obtained in a blinded fashion by 2 independent investigators.

Immunohistochemistry was performed using the Vectastain Elite ABC kit (Vector Laboratories, Burlingame, United States) and 3,3′-diaminobenzidine as staining reagent as previously described [19], [20]. Antigen unmasking was carried out by heating the sections for 20 min in 0.01 M sodium citrate (pH 6.0) at 100°C. CD3e-positive and S100a8-positive cells were detected using antibodies against CD3e (Dako, Glostrup, Denmark) and S100a8 (R&D Systems, Abingdon, United Kingdom), respectively.

### RNA Isolation and Quantitative PCR (qPCR)

Total RNA was isolated using the QIAamp RNA midi-kit (Qiagen, Venlo, the Netherlands) following the manufacturer’s protocol. Complementary DNA was synthesized from 1.5 mg RNA using M-MLV reverse transcriptase (Promega, Leiden, the Netherlands). The qPCR analysis was performed based on the intercalation of SYBR Green on an ABI Prism 7700 sequence detection system (PE Applied Biosystems, Foster City, United States) as previously described [19]. The relative mRNA expression levels were normalized against *β-Actin (Actb)* expression levels of each mouse. The sequences of all primers used for qPCR are given in Table 2. The oligonucleotide sequences were designed using OLIGO 6.22 software based on the gene sequences and purchased from Invitrogen. All primers had a melting temperature between 65°C and 66.5°C. The specificity and efficiency of all primer sets were tested, and all PCRs performed with comparable efficiencies of 95% or higher.

**Table 2:** Primer sequences used for qPCR in this study.

Target gene	Forward Primer (5′–3′)	Reverse Primer (5′– 3′)
*Actb*	GGGACCTGACGGACTAC	TGCCACAGGATTCCATAC
*Cd45*	TTTGGGAACATTACTGTGAA	TGGAGCACATGAGTCATTAG
*Cd3e*	CCAGCCTCAAATAAAAACA	TTGGCCTTCCTATTCTTG
*Tnf*	TGGCCTCCCTCTCATC	GGCTGGCACCACTAGTT
*Foxp3*	ACACCCAGGAAAGACAG	GGCAGTGCTTGAGAAAC
*Tgfb1*	AACCAAAGACATCTCACACA	GCCAGGAATTGTTGCTAT
*Ebi3*	CCCGGACATCTTCTCTCT	GAGGCTCCAGTCACTTG
*Il12a*	GCCTTGGTAGCATCTATGAG	TCGGCATTATGATTCAGAGA
*Pparg*	CAGTTTCGATCCGTAGAAGC	CCATAAAGTCACCAAAGGGC
*Sod1*	GATCGTGTGATCTCACTCTC	TTGTTTCTCATGGACCAC
*Sod3*	GAACTTCACCAGAGGGAA	GACATGGTGACAGAGCC
*Gpx1*	CCCGTGCAATCAGTTC	TTCGCACTTCTCAAACAA

### Statistical Analysis

The data are expressed as median or mean ± SEM. The data from HBPF and HAPF groups were compared using Chi-square test or Mann-Whitney test. AIN-93G group served as a reference group; therefore all parameters were also compared with this group. The data were considered statistically significant at *p*<0.05.

## Results

### Clinical Symptoms

**Figure 1:**
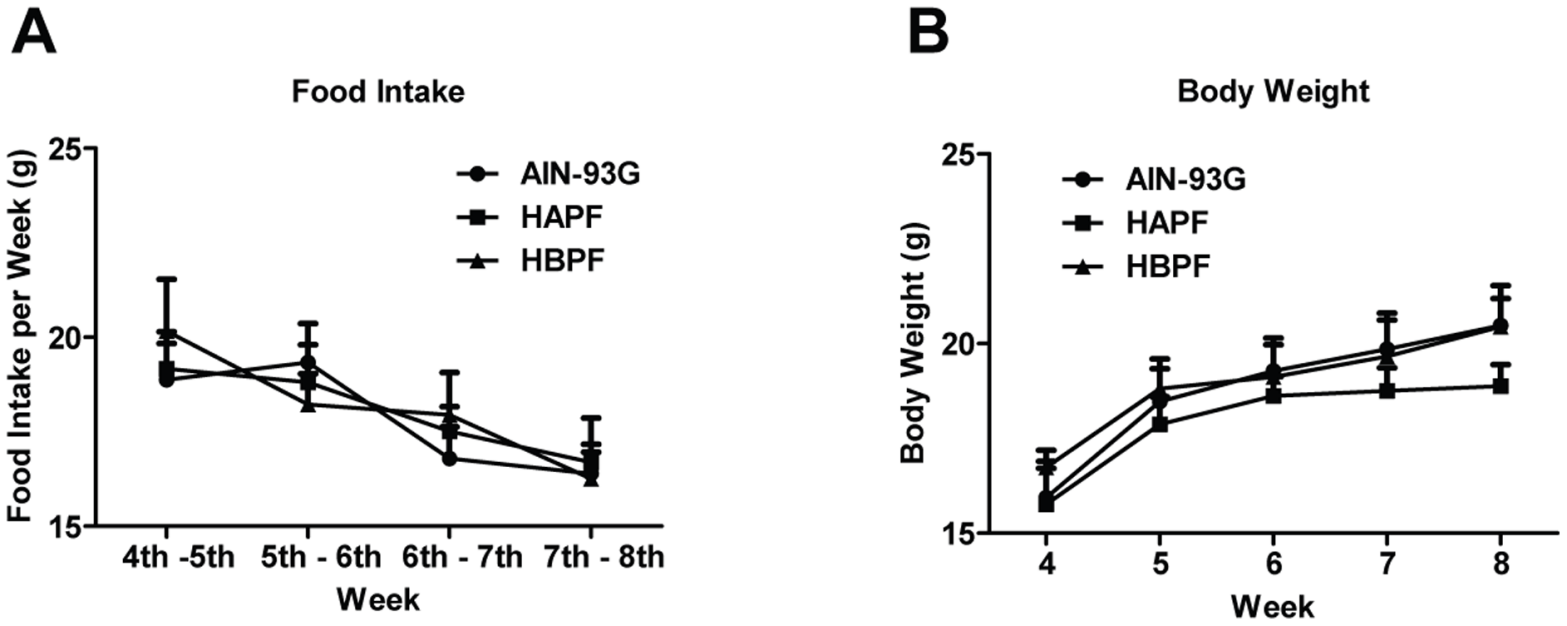
Clinical Symptoms of Muc2^−/−^ mice fed AIN-93G, HAPF or HBPF diet. Food intake (A) and body weights (B) of mice fed AIN-93G, HAPF or HBPF diet were recorded from the age of 4 weeks until the end of the study. Each group represents 6 animals in total; 3 males and 3 females.

The fats did not affect total food intake as no significant differences among the HAPF, HBPF and AIN-93G groups was observed during the study period (Figure 1A). Weight loss or growth retardation is considered as one of the major clinical symptoms of colitis, and therefore, we next compared body weights. The mean value of body weight of mice fed HAPF diet was slightly lower than that of mice fed AIN-93G or HBPF diets, but the differences were not statistically significant at any time point investigated (Figure 1B). Another clinical symptom of colitis is rectal bleeding. At the age of 8 weeks, 2 of 6 mice (33%) from the AIN-93G diet group, 3 of 6 mice (50%) from the HAPF diet group and only 1 of 6 mice (16%) from the HBPF diet group showed rectal bleeding.

### HAPF, but not HBPF, Increases Intestinal Mucosal Damage

**Figure 2:**
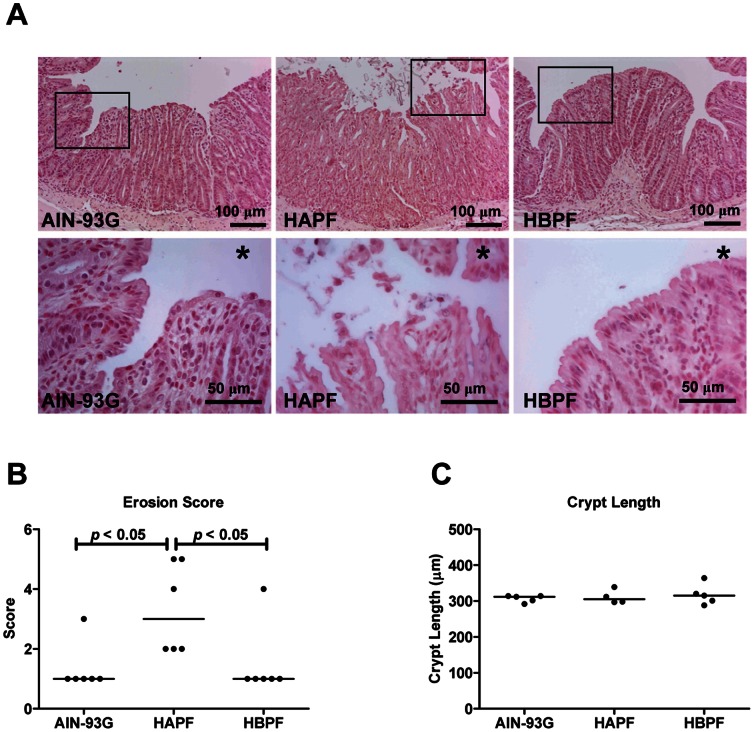
Morphology of the distal colon of Muc2^−/−^ mice fed AIN-93G, HAPF or HBPF diet. Distal colonic sections of mice fed with different diets were stained with haematoxylin and eosin, and representative sections of each diet group are shown (A). Panels with an asterisk represent a higher magnification of the surface epithelium of the distal colon. Erosion scores (B) and crypt lengths (C) within in each diet group are shown. Images are representative for all the mice in each diet group.

Ruffled, flattened and erosive epithelia were observed in the distal colon of all Muc2^−/−^ mice. However, the extent of morphological changes within the surface epithelium varied among the groups (Figure 2A). The remaining surface epithelium in the AIN-93G group consisted of approximately 20–30% normal, polarized, columnar epithelial cells, and 30–70% of the epithelial cells were ruffled and flattened. However, the remaining surface epithelium of the animals in the HAPF group showed extensive damage, and normal epithelial cells were not observed in these animals. The HBPF group exhibited similar results to those observed in the AIN-93G reference group. In line with this, the erosion score was significantly increased in the HAPF group compared with HBPF and AIN-93G groups (Figure 2B). We previously identified crypt lengthening as a site-specific marker for colitis severity in Muc2^−/−^ mice [19], [20]. Therefore we analyzed crypt lengths in Muc2^−/−^ mice fed the different diets. No significant differences were observed among the three diet groups (Figure 2C).

### HAPF and HBPF do not Alter the Mucosal T cell Influx in the Distal Colon

**Figure 3:**
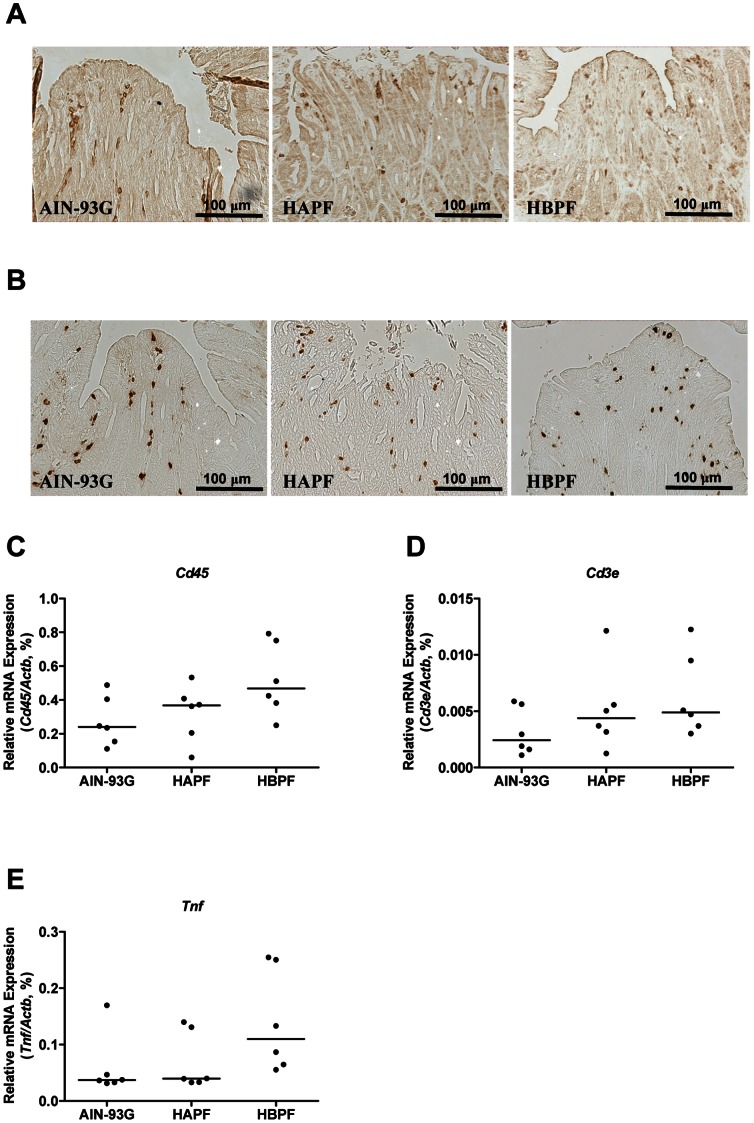
Inflammatory response in the distal colon of Muc2^−/−^ mice fed AIN-93G, HAPF or HBPF diet. Representative stainings of CD3e-positive T cells (A) and S100a8-positive cells (B) in the distal colon of each diet group are shown. Transcription of *Cd45* (C), *Cd3e* (D) and *Tnf* (E) in the distal colon of mice was analyzed using qPCR. No significant difference was observed. Each group represents 6 animals in total; 3 males and 3 females.

Following the relatively normal surface epithelial morphology in HBPF group compared with HAPF group, we next determined whether HBPF suppressed the inflammatory response in Muc2^−/−^ mice. We used the influx of CD3e-positive T cells as a marker for intestinal inflammation. In the distal colon, the amount of CD3e-positive T cells did not differ among the three diet groups (Figure 3A). We also performed immunohistochemistry for S100a8, an inflammation related protein which is mainly produced by neutrophils. Similarly, the abundance of S100a8-positive cells did not differ among the three diet groups (Figure 3B).

In addition to the immunohistochemical analysis, the mRNA expression levels of *Cd45* and *Cd3e* were also quantified. No differences were seen in the mRNA expression levels of *Cd45* and *Cd3e* among the three diet groups (Figure 3C & 3D), indicating that the total numbers of hematopoietic cells and T cells were not altered by the type of diet studied.

We next quantified the expression of the pro-inflammatory cytokine tumor necrosis factor-α (*Tnf*). There was no significant difference in *Tnf* expression levels between mice fed HBPF and HAPF diets (Figure 3E), and there were also no significant differences in the mRNA expression levels of other inflammatory markers such as inducible nitric oxide synthase 2 (*iNos2*), interleukin 1 beta (*Il1b*), interleukin 4 (*Il4*), and the Th17 signature cytokines interleukin 17 (*Il17*) and interleukin 22 (*Il22*) (data not shown).

### HBPF Enhances Regulatory T cell Response

**Figure 4:**
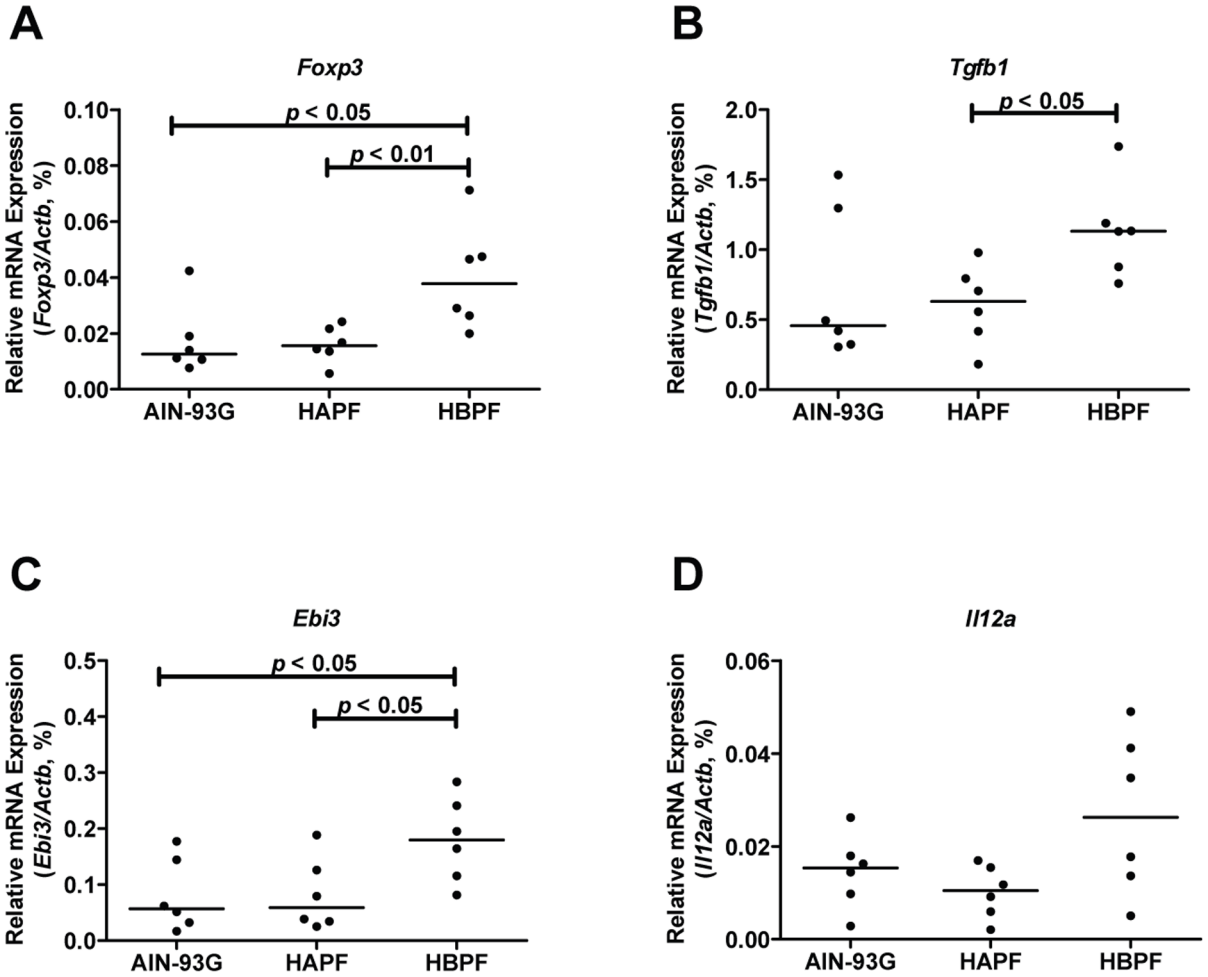
Enhanced regulatory T cell response in the distal colon of Muc2^−/−^ mice fed HBPF diet. The transcription levels of *Foxp3* (A), *Tgfb1* (B), *Ebi3* (C) and *Il12a* (D) in the distal colon of mice were analyzed using qPCR. Each group represents 6 animals in total; 3 males and 3 females.

Treg cells are shown to have immunosuppressive capacities [22], and therefore, we next determined the Treg response in the three diet groups. The *Foxp3* mRNA expression did not differ between HAPF and AIN-93G groups, but it was significantly up-regulated in HBPF group compared with HAPF and AIN-93G groups (Figure 4A), suggesting that HBPF induced an immunosuppressive Treg cell response.

Treg cells suppress the pro-inflammatory response by inhibiting effector T cells primarily through the production of cytokines, such as transforming growth factor beta 1 (Tgfb1) and interleukin 35 (IL35) [26], [27]. HBPF diet significantly up-regulated *Tgfb1* mRNA expression levels in the distal colon compared with HAPF diet (Figure 4B). IL35 is a heterodimeric cytokine composed of an interleukin 12A (IL12a) subunit and an Epstein-Barr virus induced 3 (Ebi3) subunit. Interestingly, *Ebi3* mRNA expression levels were up-regulated in the HBPF group compared with the HAPF group and the AIN-93G (Figure 4C), and *Il12a* mRNA levels trended higher in the HBPF group (Figure 4D). There were no differences in the mRNA expression levels of these Treg signature cytokines between HAPF and AIN-93G groups (Figure 4B to 4D).

### HBPF Up-regulates Proliferator-activated Receptor Gamma (PPAR-gamma) and Enzymatic Antioxidants

**Figure 5:**
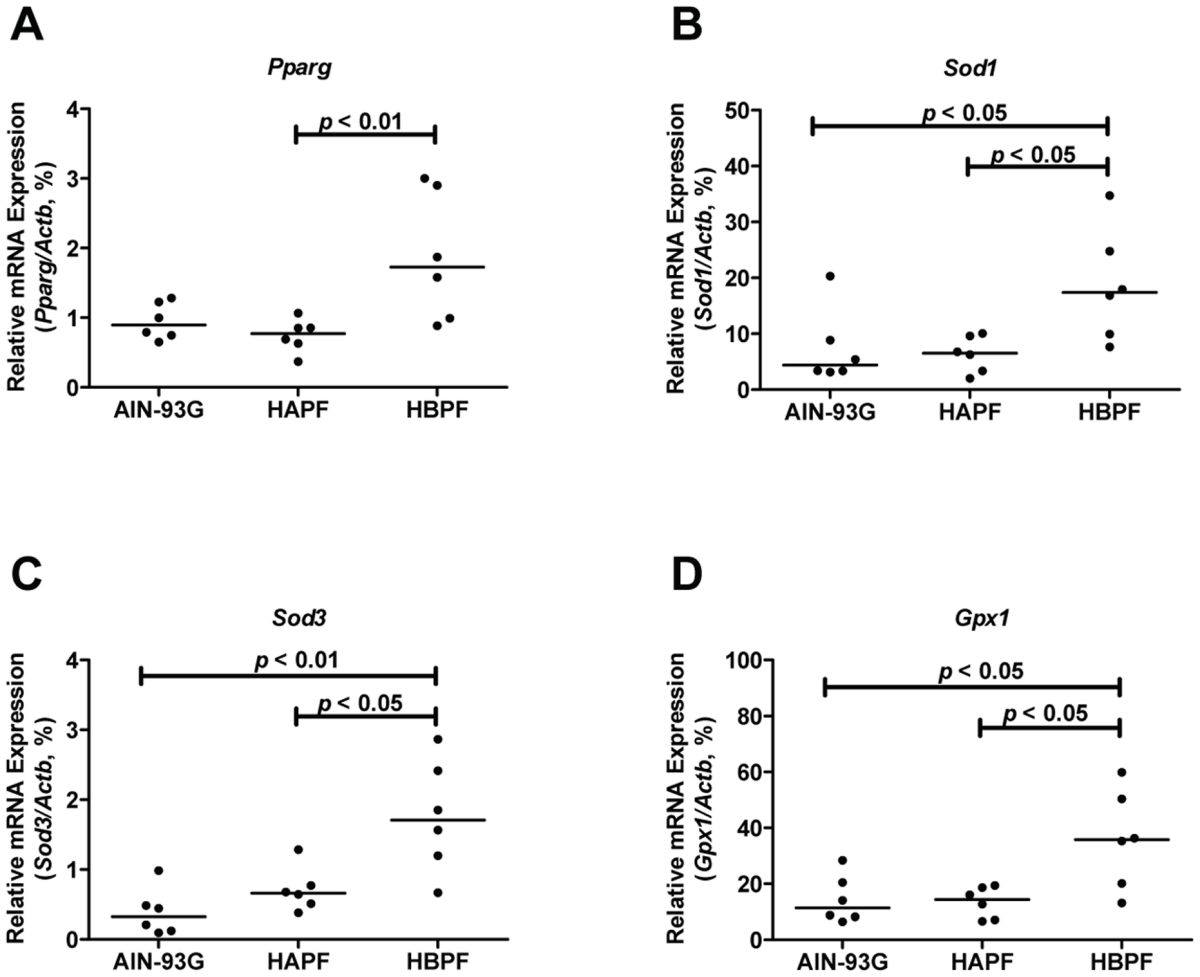
Increased *Pparg* and enzymatic antioxidants in the distal colon of Muc2^−/−^ mice fed HBPF diet. The transcription of *Pparg* (A), *Sod1* (B), *Sod3* (C) and *Gpx1* (D) in the distal colon of mice was analyzed using qPCR. Each group represents 6 animals in total; 3 males and 3 females.

PPAR-gamma is shown to prevent gut inflammation by favouring the recruitment of Treg to the mucosal inductive sites [28]. HAPF diet did not change the *Pparg* mRNA expression levels compared with AIN-93G diet. However, *Pparg* mRNA expression was significantly up-regulated in the HBPF group compared with the HAPF group, and there was a trend towards increasing *Pparg* mRNA expression levels in the HBPF group compared with the AIN-93G group (Figure 5A).

Finally, as antioxidants modulate the inflammation in IBD [29], we quantified the mRNA expression levels of enzymatic antioxidants cytoplasm superoxide dismutase 1 (*Sod1*), extracellular superoxide dismutase 3 (*Sod3*) and glutathione peroxidase 1 (*Gpx1*). The mRNA expression levels of these three antioxidant enzymes did not differ between HAPF and AIN-93G groups, but they were all up-regulated in the HBPF group (Figure 5B to 5D).

## Discussion

The HAPF and HBPF diets used in this study contain a similar amount of total palmitic acid. However, HBPF diet contains high levels of palmitic acid esterified to the sn-2 position of the triglycerides and mimics the fat composition and properties of human milk fat, while HAPF diet contains high levels of palmitic acid esterified to the sn-1,3 positions. In the current study, HAPF diet increased the incidence of erosion and mucosal damage in the distal colon of Muc2^−/−^ mice compared with the AIN-93G reference diet, but HBPF diet did not. Moreover, HBPF diet induced an immunosuppressive Treg response.

The different fat blends did not affect the total food intake in the three diet groups. However, compared with mice fed AIN-93G reference diet, which contains soybean oil as fat source, mice fed HAPF diet, which contains palm oil with high levels of alpha, alpha’-palmitate and low levels of beta-palmitate, showed significantly higher erosion scores and more intestinal mucosal damage. These data imply that HAPF diet increased the colonic mucosal damage in Muc2^−/−^ mice compared with the AIN-93G reference diet. In contrast, HBPF diet, which contains palm oil with low levels of alpha, alpha’-palmitate and high levels of beta-palmitate, significantly reduced the occurrence of epithelial erosions and improved the intestinal morphology in the distal colon compared with HAPF diet. Additionally, less mice fed HBFP diet showed rectal bleeding compared with mice fed HAPF diet (1 of 6 versus 3 of 6). There were no differences regarding the erosion score, intestinal morphology, body weight and rectal bleeding between HBPF and AIN-93G groups.

Intestinal inflammation was extensively investigated using multiple inflammation markers. The abundance of CD3e-positive T cells and S100a8-positive neutrophils was not altered among the three diet groups, and the expression of inflammation markers (e.g. *Tnf*, *iNos2*, *Il1b*, *Il4*, *Il17* and *Il22*) remained unaltered among the three diet groups. In sharp contrast, morphological signs of inflammation such as flattening of surface epithelium, detachment of surface epithelium from the basement membrane, and erosions were limited in mice fed HBPF diet compared with HAPF diet. Based on these data, one could hypothesize that in the HBPF group the pro-inflammatory response is counterbalanced by an immunosuppressive response, thereby limiting colonic damage. In line with this hypothesis are the increased levels of *Foxp3* and *Tgfb1* mRNA levels in the HBPF group, but not in HAPF and AIN-93G groups. Our data also show that IL35, a heterodimeric cytokine composed of IL12a and Ebi3 which is produced by Treg cells [27], was up-regulated by the HBPF diet group. Interestingly, we previously showed an up-regulated local immunosuppressive Treg response and no histological damage in the distal colon of Muc2^−/−^ mice before weaning [21]. However, after weaning the immunosuppressive Treg response in the distal colon had declined and colonic mucosal damage appeared in these mice. Based on this we suggest that, in our current study, the HBPF-induced immunosuppressive Treg response in Muc2^−/−^ mice limits mucosal damage and thereby colitis severity.

In line with the increased hallmarks of an immunosuppressive Treg response in HBPF group compared with HAPF and AIN-93G groups, HBPF diet also up-regulated the expression levels of *Pparg* and enzymatic antioxidants. Interestingly, PPAR-gamma has important immunoregulatory functions in the intestinal homeostasis and inflammation [30], [31], and it is an important factor controlling the accumulation and phenotype of Treg cells residing in the intestine [32]. For example, endogenous PPAR-gamma activation down-regulates effector T cell function and prevents colitis [33]. Moreover, PPAR-gamma agonist-induced Treg cells maintain a high level of Foxp3 expression [34], and deficiency of PPAR-gamma in T cells accelerates the onset of colitis and decreases the number of Treg cells [28]. It has also been documented that dietary antioxidants reduce Treg apoptosis and increase the yield of Treg cells, and therefore decrease the inflammation [35], [36]. Enzymatic antioxidants, such as superoxide dismutases, were shown to ameliorate colitis in animal models [37] and improve ulcerative colitis in patients [38]. Further, Gpx1 activity is associated with enhanced Treg cell activity in mice [39], and mice deficient in both glutathione peroxidases, Gpx1 and Gpx2, develop spontaneous colitis [40]. Therefore, we suggest that in our study the up-regulation of *Pparg* and antioxidant enzymes (*Sod1*, *Sod3* and *Gpx1*) by HBPF might contribute to the induction of an immunosuppressive Treg response, thereby controlling the inflammatory response and limiting the incidence of erosions in Muc2^−/−^ mice.

Overall, based on these data it is tempting to speculate that HAPF, a fat constituent used in regular infant formula [3], might be involved in the onset of intestinal damage in infants prone to develop intestinal inflammation such as preterm infants. Replacement of HAPF with HBPF (i.e., fat containing high levels of palmitic acid esterified to the sn-2 position), could enhance the immunosuppressive Treg response in the intestine and thereby prevent or limit intestinal inflammation. However, this is all highly speculative and should be investigated further.

In summary, our data show for the first time that HAPF diet increases the incidence of intestinal erosions and mucosal damage, while HBPF diet controls the damage by inducing an immunosuppressive Treg response in Muc2^−/−^ mice. Additionally, HBPF diet stimulates expression of *Pparg* and antioxidant enzymes (*Sod1*, *Sod3* and *Gpx*), which might be linked to the protective Treg response. Together, these data imply a crucial role for beta-palmitic acid in limiting intestinal inflammation.

## References

[1] Manson WG, Weaver LT (1997) Fat digestion in the neonate. Arch Dis Child Fetal Neonatal Ed 76: F206–211. 9175955 10.1136/fn.76.3.f206 PMC1720654

[2] Lopez-Lopez A, Lopez-Sabater MC, Campoy-Folgoso C, Rivero-Urgell M, Castellote-Bargallo AI (2002) Fatty acid and sn-2 fatty acid composition in human milk from Granada (Spain) and in infant formulas. Eur J Clin Nutr 56: 1242–1254. 12494309 10.1038/sj.ejcn.1601470

[3] de Fouw NJ, Kivits GA, Quinlan PT, van Nielen WG (1994) Absorption of isomeric, palmitic acid-containing triacylglycerols resembling human milk fat in the adult rat. Lipids 29: 765–770. 7869857 10.1007/BF02536698

[4] Teichert SA, Akoh CC (2011) Stearidonic acid soybean oil enriched with palmitic acid at the sn-2 position by enzymatic interesterification for use as human milk fat analogues. J Agric Food Chem 59: 5692–5701. 21517012 10.1021/jf200336t

[5] Pina-Rodriguez AM, Akoh CC (2009) Enrichment of amaranth oil with ethyl palmitate at the sn-2 position by chemical and enzymatic synthesis. J Agric Food Chem 57: 4657–4662. 19413361 10.1021/jf900242g

[6] Lopez-Lopez A, Castellote-Bargallo AI, Campoy-Folgoso C, Rivero-Urgel M, Tormo-Carnice R, et al (2001) The influence of dietary palmitic acid triacylglyceride position on the fatty acid, calcium and magnesium contents of at term newborn faeces. Early Hum Dev 65 Suppl: S83–94 10.1016/s0378-3782(01)00210-9 11755039

[7] Forsythe CE, French MA, Goh YK, Clandinin MT (2007) Cholesterolaemic influence of palmitic acid in the sn-1, 3 v. the sn-2 position with high or low dietary linoleic acid in healthy young men. Br J Nutr 98: 337–344. 17391563 10.1017/S0007114507704993

[8] Nelson CM, Innis SM (1999) Plasma lipoprotein fatty acids are altered by the positional distribution of fatty acids in infant formula triacylglycerols and human milk. Am J Clin Nutr 70: 62–69. 10393140 10.1093/ajcn/70.1.62

[9] Sanders TA, Filippou A, Berry SE, Baumgartner S, Mensink RP (2011) Palmitic acid in the sn-2 position of triacylglycerols acutely influences postprandial lipid metabolism. Am J Clin Nutr 94: 1433–1441. 22030225 10.3945/ajcn.111.017459

[10] Lee YS, Kang EY, Park MN, Choi YY, Jeon JW, et al (2008) Effects of sn-2 palmitic acid-fortified vegetable oil and fructooligosaccharide on calcium metabolism in growing rats fed casein based diet. Nutr Res Pract 2: 3–7. 20126357 10.4162/nrp.2008.2.1.3 PMC2815305

[11] Jeffery NM, Sanderson P, Newsholme EA, Calder PC (1997) Effects of varying the type of saturated fatty acid in the rat diet upon serum lipid levels and spleen lymphocyte functions. Biochim Biophys Acta 1345: 223–236. 9150243 10.1016/s0005-2760(96)00174-9

[12] Velcich A, Palumbo L, Selleri L, Evans G, Augenlicht L (1997) Organization and regulatory aspects of the human intestinal mucin gene (MUC2) locus. J Biol Chem 272: 7968–7976. 9065467 10.1074/jbc.272.12.7968

[13] Dharmani P, Srivastava V, Kissoon-Singh V, Chadee K (2009) Role of Intestinal Mucins in Innate Host Defense Mechanisms against Pathogens. J Innate Immun 1: 123–135. 20375571 10.1159/000163037 PMC7312850

[14] Hecht G (1999) Innate mechanisms of epithelial host defense: spotlight on intestine. Am J Physiol 277: C351–358. 10484321 10.1152/ajpcell.1999.277.3.C351

[15] Hollingsworth MA, Swanson BJ (2004) Mucins in cancer: protection and control of the cell surface. Nat Rev Cancer 4: 45–60. 14681689 10.1038/nrc1251

[16] Johansson ME, Ambort D, Pelaseyed T, Schutte A, Gustafsson JK, et al (2011) Composition and functional role of the mucus layers in the intestine. Cell Mol Life Sci 68: 3635–3641. 21947475 10.1007/s00018-011-0822-3 PMC11114784

[17] Johansson ME, Phillipson M, Petersson J, Velcich A, Holm L, et al (2008) The inner of the two Muc2 mucin-dependent mucus layers in colon is devoid of bacteria. Proc Natl Acad Sci U S A 105: 15064–15069. 18806221 10.1073/pnas.0803124105 PMC2567493

[18] Velcich A, Yang W, Heyer J, Fragale A, Nicholas C, et al (2002) Colorectal cancer in mice genetically deficient in the mucin Muc2. Science 295: 1726–1729. 11872843 10.1126/science.1069094

[19] Van der Sluis M, De Koning BA, De Bruijn AC, Velcich A, Meijerink JP, et al (2006) Muc2-deficient mice spontaneously develop colitis, indicating that MUC2 is critical for colonic protection. Gastroenterology 131: 117–129. 16831596 10.1053/j.gastro.2006.04.020

[20] Lu P, Burger-van Paassen N, van der Sluis M, Witte-Bouma J, Kerckaert JP, et al.. (2011) Colonic gene expression patterns of mucin muc2 knockout mice reveal various phases in colitis development. Inflamm Bowel Dis. 10.1002/ibd.21592 21910166

[21] Burger-van Paassen N, van der Sluis M, Bouma J, Korteland-van Male AM, Lu P, et al (2011) Colitis development during the suckling-weaning transition in mucin Muc2-deficient mice. Am J Physiol Gastrointest Liver Physiol 301: G667–678. 21700902 10.1152/ajpgi.00199.2010

[22] Sakaguchi S (2005) Naturally arising Foxp3-expressing CD25+CD4+ regulatory T cells in immunological tolerance to self and non-self. Nat Immunol 6: 345–352. 15785760 10.1038/ni1178

[23] Boden EK, Snapper SB (2008) Regulatory T cells in inflammatory bowel disease. Curr Opin Gastroenterol 24: 733–741. 19125486 10.1097/mog.0b013e328311f26e

[24] Reeves PG (1997) Components of the AIN-93 diets as improvements in the AIN-76A diet. J Nutr 127: 838S–841S. 9164249 10.1093/jn/127.5.838S

[25] Renes IB, Verburg M, Van Nispen DJ, Taminiau JA, Buller HA, et al (2002) Epithelial proliferation, cell death, and gene expression in experimental colitis: alterations in carbonic anhydrase I, mucin MUC2, and trefoil factor 3 expression. Int J Colorectal Dis 17: 317–326. 12172925 10.1007/s00384-002-0409-4

[26] Bommireddy R, Doetschman T (2007) TGFbeta1 and Treg cells: alliance for tolerance. Trends Mol Med 13: 492–501. 17977791 10.1016/j.molmed.2007.08.005 PMC2805009

[27] Collison LW, Workman CJ, Kuo TT, Boyd K, Wang Y, et al (2007) The inhibitory cytokine IL-35 contributes to regulatory T-cell function. Nature 450: 566–569. 18033300 10.1038/nature06306

[28] Guri AJ, Mohapatra SK, Horne WT, 2nd, Hontecillas R, Bassaganya-Riera J (2010) The role of T cell PPAR gamma in mice with experimental inflammatory bowel disease. BMC Gastroenterol 10: 60. 20537136 10.1186/1471-230X-10-60 PMC2891618

[29] Rezaie A, Parker RD, Abdollahi M (2007) Oxidative stress and pathogenesis of inflammatory bowel disease: an epiphenomenon or the cause? Dig Dis Sci 52: 2015–2021. 17404859 10.1007/s10620-006-9622-2

[30] Mohapatra SK, Guri AJ, Climent M, Vives C, Carbo A, et al (2010) Immunoregulatory actions of epithelial cell PPAR gamma at the colonic mucosa of mice with experimental inflammatory bowel disease. PLoS One 5: e10215. 20422041 10.1371/journal.pone.0010215 PMC2857885

[31] Bento AF, Marcon R, Dutra RC, Claudino RF, Cola M, et al (2011) beta-Caryophyllene inhibits dextran sulfate sodium-induced colitis in mice through CB2 receptor activation and PPARgamma pathway. Am J Pathol 178: 1153–1166. 21356367 10.1016/j.ajpath.2010.11.052 PMC3070571

[32] Cipolletta D, Feuerer M, Li A, Kamei N, Lee J, et al (2012) PPAR-gamma is a major driver of the accumulation and phenotype of adipose tissue Treg cells. Nature 486: 549–553. 22722857 10.1038/nature11132 PMC3387339

[33] Hontecillas R, Bassaganya-Riera J (2007) Peroxisome proliferator-activated receptor gamma is required for regulatory CD4+ T cell-mediated protection against colitis. J Immunol 178: 2940–2949. 17312139 10.4049/jimmunol.178.5.2940

[34] Lei J, Hasegawa H, Matsumoto T, Yasukawa M (2010) Peroxisome proliferator-activated receptor alpha and gamma agonists together with TGF-beta convert human CD4+CD25- T cells into functional Foxp3+ regulatory T cells. J Immunol 185: 7186–7198. 21057085 10.4049/jimmunol.1001437

[35] Ma X, Hua J, Mohamood AR, Hamad AR, Ravi R, et al (2007) A high-fat diet and regulatory T cells influence susceptibility to endotoxin-induced liver injury. Hepatology 46: 1519–1529. 17661402 10.1002/hep.21823

[36] Tan PH, Sagoo P, Chan C, Yates JB, Campbell J, et al (2005) Inhibition of NF-kappa B and oxidative pathways in human dendritic cells by antioxidative vitamins generates regulatory T cells. J Immunol 174: 7633–7644. 15944264 10.4049/jimmunol.174.12.7633

[37] Segui J, Gironella M, Sans M, Granell S, Gil F, et al (2004) Superoxide dismutase ameliorates TNBS-induced colitis by reducing oxidative stress, adhesion molecule expression, and leukocyte recruitment into the inflamed intestine. J Leukoc Biol 76: 537–544. 15197232 10.1189/jlb.0304196

[38] Suzuki Y, Matsumoto T, Okamoto S, Hibi T (2008) A lecithinized superoxide dismutase (PC-SOD) improves ulcerative colitis. Colorectal Dis 10: 931–934. 18294268 10.1111/j.1463-1318.2008.01487.x PMC2659364

[39] Tsai PY, Ka SM, Chang JM, Chen HC, Shui HA, et al (2011) Epigallocatechin-3-gallate prevents lupus nephritis development in mice via enhancing the Nrf2 antioxidant pathway and inhibiting NLRP3 inflammasome activation. Free Radic Biol Med 51: 744–754. 21641991 10.1016/j.freeradbiomed.2011.05.016

[40] Esworthy RS, Aranda R, Martin MG, Doroshow JH, Binder SW, et al (2001) Mice with combined disruption of Gpx1 and Gpx2 genes have colitis. Am J Physiol Gastrointest Liver Physiol 281: G848–855. 11518697 10.1152/ajpgi.2001.281.3.G848

